# Investigating Effects of Small-Group Student Talk on the Quality of Argument in Chinese Tertiary English as a Foreign Language Learners’ Argumentative Writing

**DOI:** 10.3389/fpsyg.2022.868045

**Published:** 2022-06-14

**Authors:** Hui Helen Li, Lawrence Jun Zhang

**Affiliations:** ^1^School of Foreign Languages, Wuhan University of Technology, Wuhan, China; ^2^Faculty of Education and Social Work, University of Auckland, Auckland, New Zealand

**Keywords:** quality of argument, small-group student talk, sociocultural theory, Chinese tertiary EFL learners, argumentative writing

## Abstract

Previous studies have offered a rationale for engaging students in small-group student talk for the planning of L2 individual writing. To further investigate whether such talk effectively promotes the quality of argument in the context of Chinese tertiary EFL learners’ argumentative writing and whether such effects could be retained, the current study adopted a quasi-experimental design with a pretest, a posttest, and a delayed posttest in two intact EFL classes. The performance of the intervention group and the comparison group were scrutinized to examine the effects of the intervention. The analytic scores on six components of the writing task (claim, data, counterargument claim, counterargument data, rebuttal claim, and rebuttal data) and the holistic writing scores cumulated of all these components were measured to see the immediate and sustained effects. Significant changes of the holistic scores in both the immediate posttest and the delayed posttest indicated that such small-group student talk enabled students in the treatment class to achieve better performance in the overall quality of argumentation compared with those in the comparison class. Statistical analyses revealed immediate and sustained effects of small-group student talk on the quality of counterargument claim, counterargument data, and rebuttal claim. Counterargument claim was the only element in which students in both classes made significant improvement, but the treatment class demonstrated a larger effect size. No discernible differences were found either between or within the treatment class and the comparison class with respect to the quality of claim, data, and rebuttal data across tests. Possible explanations concerning the findings and limitations of the study were discussed.

## Introduction

### Theoretical Framework

Writing as the most challenging skill for learners in the process of learning a foreign language (Zhang, 2013; Zhao and Zhang, 2022) has been viewed as a meaning-making activity that takes place as part of a social and cultural scene (Parr et al., 2009; Parr and Wilkinson, 2016) to accomplish inherently social goals (Bazerman, 2016). From the perspective of Sociocultural Theory (Vygotsky, 1978), the interactions and collaborations during the socially situated writing processes can scaffold the internalization of cognitive and linguistic skills and thus lead to improved writing performance (Lockhart and Ng, 1995). In this vein, the social discussions and collaborations with others scaffold the individual to construct knowledge first externally and then internally within the individual’s zone of proximal development (ZPD)—the gap between the individual’s actual and potential developmental levels (Vygotsky, 1978; see also Ortega, 2009, for a discussion). Besides ZPD, “collective scaffolding” (Donato, 1994) enables an expert to scaffold a novice in unequal conditions and supports a peer to scaffold one another in equal conditions (Walqui, 2006; Kibler, 2017). In this way, supportive collaborations can be established by means of speech in which the novice or the peer can “participate, and extend current skills and knowledge to higher levels of competence” (p. 40). To be specific, students in the writing classroom can collectively scaffold each other to reach a higher level of writing development in the processes of negotiating writing tasks by means of small-group interactions rather than think alone by themselves (Neumann and McDonough, 2015; Li et al., 2020). The constructs of ZPD and collective scaffolding in Sociocultural Theory solidly theorize the employment of small-group student talk for developing students’ individual writing.

### Small-Group Student Talk

Strijbos et al. (2004) classified three frequently used types of groups for collaborative interactions: Dyads (two members), small groups (three to six members), and large groups (seven or more). Based on the fact that there are large classes in the Chinese tertiary EFL context, it is thus both difficult for the teacher to manage too many dyads in the writing classroom and also challenging for the teacher to guarantee opportunities for each student to talk in groups of seven or more members within the limited class time. More importantly, the classroom desks and chairs in most of the Chinese universities are not movable, so groups must sit close to each other, which raises noise issues if small groups with fewer students in each group are formed and all groups discuss at the same time. Therefore, in this study, small-group student talk refers to the meaningful interactions among small groups with six students formed by the self-selection method during which students talk about writing tasks before they proceed to their individual writing (Li et al., 2020; Li and Zhang, 2021a). According to Li et al. (2020), students who are engaged in such talk shared the responsibility for managing the group as well as the ongoing process of the writing task instead of directing someone as a fixed group leader throughout the whole procedure. To this end, six students interacting in a small group offers students opportunities to assume various roles to well manage the group and the writing task.

Aligning with this definition, small-group student talk occurs during planning, the preparation for the action of writing, which is believed to be crucial and indispensable to produce written texts (Ellis and Yuan, 2004) because a planned language output will most possibly push learners to reach their potential developmental levels of language use (Mirazi and Mahmoudi, 2016). Within the Sociocultural Theory framework, meaningful verbal interactions among small groups that occur during planning can foster writing development, knowledge co-construction, and social communication (Fernández Dobao, 2012, 2014; Li and Zhu, 2013; Li and Zhang, 2021a). Specifically, small-group student talk enables all participants to agree or disagree with one another by sharing opinions and evidence on the writing topic, which offers all participants a platform to generate and evaluate ideas and evidence and helps them select and organize those into a writing plan (Neumann and McDonough, 2015; Li and Zhang, 2021b). Additionally, small-group student talk also facilitates students to collectively scaffold one another to negotiate meaning (Storch, 2019), produce writing ideas (Watanabe and Swain, 2007; Shehadeh, 2011), solve linguistic problems in writing (Fernández Dobao, 2012, 2014), promote peer collaboration, and practice for writing development (Storch and Wigglesworth, 2007; Wigglesworth and Storch, 2009), among other things.

Considering that verbal interaction is one of the most extensively-used ways, also the most effective form for knowledge construction (Palincsar and Brown, 1989; Fernández Dobao, 2007), students in the writing classroom should be encouraged to actively talk with their peers so that those who are scaffolded by peers and engaged in small-group student talk have chances to discuss argumentative writing tasks collaboratively, bridge the gap between what each student knows and what they can know together, and thus co-construct a greater knowledge for writing development as a group than any of the group members would do on their own. In other words, when offered opportunities to engage in small-group student talk for the planning of their subsequent individual argumentative writing tasks, students can collectively scaffold each other for their ZPDs to be triggered and kept as active as possible until they accomplish the argumentative writing tasks which are comparatively more difficult when they do them alone (Antón and Dicamilla, 1999; Ohta, 2000). In the process of doing so, collective scaffolding can be gradually reduced as the student becomes more competent in performing the task. When the student is eventually able to accomplish the argumentative task individually, the effect of the collective scaffolding is considered retained. Underpinned by such a rationale, it is important to exploit the benefits of small-group student talk while maintaining an emphasis on individual argumentative writing development.

### Argumentative Writing and Quality of Argument

Argumentative writing, having been viewed as the most demanding writing task (Grabe and Kaplan, 1996; Siregar et al., 2021; Zhang, 2021; Zhang and Cheng, 2021) and a broadly recognized assessment for L2 learners’ writing proficiency (Teng and Zhang, 2020), aims to convince readers to accept the writer’s point of view by clearly stating a claim, selecting evidence to support such a claim, recognizing counterarguments, responding to opposing claims, and finally reaching a conclusion in a logically organized way (Nippold and Ward-Lonergan, 2010). Since to comprehend, evaluate, and construct written arguments is an essential skill for academic learning at various levels (Wolfe et al., 2009), thus it is crucial to introduce L2 learners to argumentative writing and familiarize them with the basic terms and a comprehension of the elements of argument together with the processes through which examination of reasoning becomes the burgeoning of a claim (Heidari, 2019).

In the context of the current study, an “argument” is defined, according to Toulmin (1958), as a set of claims, one of which (the primary claim or conclusion) is meant to be supported by the rest (the reasons or premises) (Zainuddin and Rafik-Galea, 2016). Initially, the Toulmin model of argument (Toulmin, 1958) only included three elements: claim, data, and warrant. Later in Toulmin, 2003 extended his original model by adding some second-level elements: qualifier, backing, and rebuttal. Since then, a standard Toulmin model of argument has been viewed as the inclusion of six elements: claim (an arguable assertion), data (the evidence to support and justify the claim), warrant (the reasoning that connects data and claim), backing (the assumptions that reinforce the warrant), qualifier (the strength conferred by the warrant), and rebuttal (the conditions in which the claim does not hold true). The overall quality of argumentative writing is more related to the quality of argument than the mere presence of the argument elements (Paek and Kang, 2017; Özdemir, 2018; Hamam, 2020; Osman and Januin, 2021). Specifically, a good argumentative writing is not the one that only focuses on “the surface structure, or the shell of the argument” (Stapleton and Wu, 2015, p. 12) or that overemphasizes on structural elements of argument at the expense of quality of logic and evidence (Macagno and Konstantinidou, 2013; Abdollahzadeh et al., 2017). Instead, it is the one with a clear claim backed up by relevant and sufficient data as well as counterarguments challenged by effective rebuttals (Qin, 2020). In other words, a good argumentative writing integrates both the surface structure (i.e., essential elements including claims, counterclaims, rebuttals, and their accompanied data) and the substance (i.e., the quality of reasoning) (Ho, 2011; Uysal, 2012; Stapleton and Wu, 2015; Qin, 2020; Siregar et al., 2021; Sundari and Febriyanti, 2021; Zhang et al., 2021). As an essential marker of critical thinking and a significant feature of argumentative writing (Wolfe, 2011; Hirvela, 2017), quality of argument has been measured with an increasing use of the Toulmin model that has gained broad acknowledgment in accounting for the various elements marking the progress of an argument (Qin, 2020) and has been widely used to assess the quality of argumentative writing (Yeh, 1998; Qin and Karabacak, 2010; Liu and Stapleton, 2014; Stapleton and Wu, 2015; Qin, 2020; Zhang and Zhang, 2021a,b).

### Effects of Small-Group Student Talk on L2 Argumentative Writing

In the recent past, research evidence in L2 documenting small-group student talk has reported mixed findings on its effects on L2 argumentative writing. One related line of research claimed positive effects by measuring linguistic features (i.e., complexity, accuracy, fluency, subordination) and/or analytic ratings (e.g., content, organization, language use, grammar, mechanics, etc.) (Shin, 2008; Pu, 2010; Neumann and McDonough, 2015; McDonough et al., 2019; Li and Zhang, 2021b). For example, Shin (2008) investigated such effects through the comparison of individual and pair-group collaborative planning of English learners in a Korean university which claimed that the planning with pair-group discussions achieved significantly higher scores than those without it in the individual planning group on all five analytic measures (content, organization, language in use, grammar, and mechanics). In a similar vein, Pu (2010) investigated the effects of prewriting discussions among groups with three students by taking the factor of different languages into consideration. He assigned 24 first-year Chinese English major students into four groups, namely, the Chinese L1 group, English L2 group, Chinese L1 and English L2 group, and the individual planning group, and measured the language quality of students’ argumentative texts written under these four conditions in terms of CAF (complexity, accuracy, and fluency). His study corroborated the effectiveness of small-group student talk given that the argumentative essays written by the English L2 group were much better in language quality with fewer errors and higher syntactical complexity than those of the other three groups. Likewise, McDonough et al. (2019), after exploring the effects of pair-group prewriting discussions in a Thai university, pointed out that although there was no significant difference for complexity measures (coordination and subordination), the texts written by students engaged in such discussions were more accurate and received higher ratings (content, organization, grammar, and vocabulary) than those written during individual planning. The findings regarding analytic ratings in these two studies were further supported by Li and Zhang (2021b), who probed the effects of the structured small-group student talk on Chinese university EFL students’ individual writing by measuring the holistic and analytic ratings of content, organization, vocabulary, language use, and mechanics in students’ written texts.

However, another line of related research has presented inconsistent findings. For instance, Shi (1998) examined adult international students’ opinion essays written independently under the conditions of peer-led pair-group talk, teacher-led pair-group talk, and no talk prior to writing and found that students wrote longer texts in the condition of no talk, shorter texts after teacher-led pair-group talk, and texts with a greater variety of verbs after peer-led pair-group talk. Her study adopted the Hamp-Lyons 9-point band scale (Hamp-Lyons, 1991), which consists of a global scale for general scores and a profile scale to measure communicative quality, organization, argumentation, linguistic accuracy, and linguistic appropriacy. Using this two-part scale for assessment, she concluded that talking versus no talking prior to writing had no noticeable effect on the scores of students’ individual writing. Another more recent study (Mirazi and Mahmoudi, 2016) adopted a quasi-experimental design to explore the effects of planning type (pair-group collaborative planning vs. individual planning) and gender on EFL learners’ writing quality by measuring the five components of content, organization, vocabulary, language use, and mechanics. Their study revealed that gender had no impact on planning type in relation to learners’ writing ability. Moreover, conflicting with Shi’s (1998) results, this study advocated that the individual planning groups had outperformed the collaborative planning groups in regard to students’ overall writing ability. Similarly, McDonough et al. (2018) examined Thai students’ English texts written under conditions of collaborative writing (writing together with a partner), collaborative prewriting (discuss with a partner during planning but write individually), and no collaboration. The study concluded that collaborative prewriting did not lead to any differences in accuracy as compared to no collaboration texts and no significant differences were found in students’ texts written under these three conditions regarding the analytic ratings (content, organization, and language). In addition, collaborative texts were more accurate than the collaborative prewriting and no collaboration texts, while the latter two contained more subordination. In another study, McDonough and De Vleeschauwer (2019), who compared the effects of collaborative planning (discuss with a partner during planning but write individually) and individual prewriting (plan alone by oneself) on Thai EFL learners’ writing development, revealed that individual planning resulted in higher analytic ratings (content, organization, and vocabulary), while collaborative planning led to improved accuracy, and no significant differences in coordination or subordination was found between these two types of planning. Their findings further evidenced that using small-group student talk for planning may not have any advantages for overall writing quality as compared to individual planning.

In summary, recent related studies addressing the effects of small-group student talk used during the planning of L2 argumentative writing have reported inconsistent or mixed findings. Besides, no studies have specifically investigated whether such talk has sustained effects on L2 individual argumentative writing. In addition, except Qin (2020) who particularly assessed the quality of argument of United Arab Emirates university students’ individual argumentative writing using Stapleton and Wu’s (2015) rubric, the majority of prior studies mainly dwelled on the measurement of linguistic features (e.g., complexity, accuracy, fluency, subordination) and/or analytic ratings (e.g., content, organization, language use, grammar, mechanics, etc.) of L2 argumentative texts, which neglected to evaluate the quality of argument—an essential marker of critical thinking and a powerful predictor of good argumentative writing (Wolfe, 2011; Ong and Zhang, 2013; Hirvela, 2017; Paek and Kang, 2017; Huang and Zhang, 2020). According to Stapleton and Wu (2015) and Qin (2020), the quality of argument is meant to be measured from both the presence of structural elements (i.e., essential elements including claims, counterclaims, rebuttals, and their accompanied data) and the quality of substance (i.e., the quality of reasoning). Evaluating the quality of argument in L2 argumentative writing is important not only because good quality of argument is considered significant for effective communication (Nussbaum and Schraw, 2007), but also because, as Qin and Karabacak (2010) argued, doing so can inform the design of instructional materials and the planning of classroom activities for L2 argumentative writing instruction. More importantly, Qin (2020) has further pointed out that it is insufficient to just instruct students to include these structural elements in their writing. In fact, more focus should be put on facilitating students to understand the different quality of these elements so that they can obtain the ability to evaluate various propositions and thus to develop their own arguments.

To resolve these uncovered issues and better understand using small-group student talk for the planning of L2 argumentative writing, this study aimed to adopt a quasi-experimental design (Creswell, 2014) implementing a pretest, a posttest, and a delayed posttest to measure the effects of such talk on the quality of argument in Chinese tertiary EFL students’ individual argumentative writing. As such, this study set out to answer the following two questions:

(1) Does small-group student talk enhance the quality of argument in Chinese tertiary EFL students’ argumentative writing regarding analytic (i.e., claim, data, counterargument claim, counterargument data, rebuttal claim, rebuttal data) and holistic scores?
(2) Is there any difference in the effect of planning with small-group student talk and that without it on students’ claim, data, counterargument claim, counterargument data, rebuttal claim, rebuttal data, and overall scores?

## Materials and Methods

### Participants and Context

The participants were 48 undergraduate students in their second year of study who were conveniently sampled (Creswell, 2014) from a School of Foreign Languages at a large public comprehensive university in Central China. All these students were admitted into the English Language and Literature Program as intermediate-level language learners by the School of Foreign Languages of the sampled university and had finished the same university coursework before their admissions into two intact classes of the same required *English Writing* course. These two classes were randomly assigned into a treatment class and a comparison class with 24 students in each. The treatment class had 19 female students and 5 male students between the ages of 18 and 20 (*M* = 19.3, *SD* = 0.76). The comparison class had 16 female students and 8 male students between the ages of 18 and 21 (*M* = 19.5, *SD* = 0.93).

The compulsory *English Writing* course spanned two semesters in the sampled university, which was designed to develop students’ competence in English writing of narrative, expository and argumentative essays and enhance their critical thinking ability (e.g., Huang and Zhang, 2020; Rahimi and Zhang, 2021). The course was instructed by an associate professor of English language and literature, who had 5 years of experience in EFL writing instruction. In the first semester, the course targeted to instruct students to write paragraphs as well as narrative and expository essays. In the second semester, its focus moved to teach students to write argumentative essays and train them to think logically and rationally about what they would be discussing and writing. The current study was conducted in the second semester for its purpose was to measure the quality of argumentation in Chinese tertiary EFL students’ individual argumentative writing.

### Materials and Design

#### Writing Tasks

The current study consisted of six structured writing task handouts (see Appendix A). All these writing tasks were chosen from the battery of China’s National English As a Foreign Language Test—Test for English Majors – Band 4 (TEM-4). Specifically, the writing tasks for pre-, post-, and delayed post-tests remained the same throughout the quasi-experiment, which is about whether the development of intelligent machines will make human brains lazy (2017 TEM-4). The writing tasks for interventions 1–5 are respectively about whether college students should hire helpers to clean their dormitories (2010 TEM-4), whether private car owners should be taxed for environmental pollution (2011 TEM-4), whether English major students should study Mathematics (2014 TEM-4), whether tourism will bring harm to the environment (2009 TEM-4), and whether it is wise to make friends online (2007 TEM-4). TEM-4 is a nationally standardized test that is taken annually by Chinese tertiary English major students at the second semester of their second year, which guaranteed the high validity and reliability of the writing tasks (Li and Zhang, 2021b). This test is designed particularly to measure whether Chinese tertiary English major students meet the required proficiency levels of English language as specified in the National College English Teaching Syllabus for English Majors (NACFLT, 2004a; cited in Jin and Fan, 2011). It contains six parts, including dictation, listening comprehension, language usage, cloze, reading comprehension, and writing. The writing section is vital for it comprises 20% of the total score of 100. Selecting the argumentative writing tasks from the TEM-4 battery provided students with opportunities to prepare for the test, which is good for arousing students’ interest and boosting their enthusiasm to participate in this study. Previous studies (Neumann and McDonough, 2015; McDonough et al., 2019; Li et al., 2020; Li and Zhang, 2021a) have proved that, compared with the naturally occurring peer group talk, structured writing tasks were more beneficial and effective for engaging L2 learners in critical evaluation of the ideas they generated and the organization they planned to make and thus helped develop L2 learners’ argumentative writing. In this sense, an extra part, modified from Neumann and McDonough (2015), was incorporated in the writing task handouts. It included three parts, respectively, stating a viewpoint to show agreement or disagreement with the writing topic, producing and evaluating ideas and evidence that support and oppose the stated viewpoint, and selecting and organizing ideas and evidence into a writing plan.

#### Writing Rubric

To measure the quality of argument in students’ argumentative writing, Stapleton and Wu’s (2015) *Analytic Scoring Rubric for Argumentative Writing* (see Appendix B) was used. This rubric was modified from the original Toulmin model of argument structure and several other researchers’ work (Nussbaum et al., 2005; Nussbaum and Schraw, 2007; Qin and Karabacak, 2010; Zhang and Zhang, 2021b) in an attempt to integrate “the assessment of both argumentative structural elements and the quality of reasoning, one of the few in the field of L2 argumentative writing assessment” (Qin, 2020, p. 230). It was specifically constructed to measure the quality of argument from both structural and substance levels because “for an argumentative essay to be persuasive, not only must it follow surface structure by including alternative viewpoints and showing their weaknesses, but it must also support claims with good quality reasons that convince others” (Stapleton and Wu, 2015, p. 22).

The rubric uses the surface structure based on Toulmin-like elements as the organizing principle for the rows. Meanwhile, it also includes the quality of the supporting reasons or evidence as the organizing principle with embedded descriptors in the columns. Altogether, there are six elements in the rows, respectively, claim (assertion in response to a writing topic), data (evidence to support claims), counterargument claim (opposing assertion that opposes the writer’s main claim), counterargument data (evidence to support the counterargument claim), rebuttal claim (assertion to refute the counterargument), and rebuttal data (evidence to support rebuttal claim). Scoring for these six elements breaks down from a 100-point scale. To assess the higher level of critical thinking and argumentation skills that enable the generation of counterargument claim and rebuttal claim, increased scores are given to these elements. Specifically, they are differentially weighted from a scale of 0–5 for claim and a scale of 0–10 for categories of counterargument claim and rebuttal claim, together with a scale of 0, 10, 15, 20, and 25 for categories of data, counterargument data, and rebuttal data (see Appendix B).

There were two reasons for employing this rubric to measure quality of argument. To start with, besides the assessment of argumentative structural elements, this rubric also enables the evaluation of substance (i.e., quality of reasoning), which is considered paramount because the analysis of structural elements without considering the strength of evidence used is not enough to assess the quality of argument (Paek and Kang, 2017; Zhang, 2018; Qin, 2020). Furthermore, since such a rubric allows graded evaluation and provides descriptors that are as inclusive as possible of the main features of good argumentative writing (Stapleton and Wu, 2015), it is thus convenient for researchers to facilitate the process of scoring.

#### Research Design

As frequently used in educational contexts which is constructed from situations that already exist in the real world (Campbell and Stanley, 1963), a quasi-experimental design (Creswell, 2014) with a pretest, a posttest, and a delayed posttest (each in 40 min) was adopted to compare the texts written independently by Chinese tertiary EFL students that were already enrolled in two parallel classes which were later randomly assigned by the first researcher into a treatment class and a comparison class.

Prior research (Shi, 1998; Neumann and McDonough, 2015; McDonough and De Vleeschauwer, 2019; Li et al., 2020; Li and Zhang, 2021a) which administered 20 min for the small-group student talk and 40 min for individual writing claimed that students could produce sufficient talk in group discussions in 20 min and drafted texts of reasonable length within 40 min. Therefore, the current study decided to administer 20 min for the prewriting small-group student talk and 40 min for the subsequent individual writing. Specifically, before proceeding to their 40 min of individual writing, students in the treatment class followed collaborative planning with 20 min of small-group student talk, while those in the comparison class planned individually for 20 min without such talk. The independent variable of the study was small-group student talk, which was operationalized in terms of the 20 min discussion of structured writing tasks in small groups of six students in the treatment class. Due to the benefits of the self-selection method which effectively facilitates participants in group collaboration with a higher sense of goal commitment and group accomplishment (Chapman et al., 2006), self-selected small groups of six students were formed in the treatment class. The dependent variable was quality of argument, which was operationalized in terms of students’ argumentative texts and measured using Stapleton and Wu’s (2015) rubric.

#### Intervention

During the quasi-experimental study, both the treatment class and the comparison class met the aforementioned instructor who mainly employed a genre approach for the *English Writing* course in a regular university classroom setting. Both classes had two 45-min class periods in each week with a total of 32 periods in a 16-week semester. Following the same teaching syllabus and plan required by the School of Foreign Languages of the selected university, both classes used a theme-based textbook^1^ that aimed to teach students how to write argumentative essays and develop their critical thinking abilities.

Students in both classes participated in the pretest, posttest, and delayed posttest respectively before, at the end of, and 4 weeks after the intervention sessions. Following a practice session in Week 2, five intervention sessions were successively carried out in the treatment class every other week from Week 3 to Week 11. Each intervention was administered with 20 min prewriting small-group student talk and 40 min subsequent individual writing of the tasks chosen from the TEM-4 battery and structured by Neumann and McDonough’s (2015) additional section that intends to promote a production of arguments and evidence. Meanwhile, in the comparison class, students were asked to plan alone for 20 min and then proceed to their 40 minutes’ individual writing of the same tasks mentioned above. All these three tests and intervention sessions were mainly conducted by the first researcher of this study. In order to obtain students’ real EFL writing performance, no external resources or help were allowed in each test. Apart from that, the writing task handouts and time and procedures of each test were kept constant in both classes.

In both the treatment and comparison classes, the instructor taught the same contents and maintained the same schedules throughout the semester. She did not intervene in students’ discussions during the intervention sessions of the treatment class. Nor did she interrupt students’ individual planning in the comparison class. Instead, she offered her assistance only when students specifically asked for it.

### Data Collection Procedures

A pretest was administered to both classes in the first week. In the following week, students in the treatment class self-selected group members and formed into four groups of six students. To avoid the impact caused by changing group members, each group was told to remain unchanged during the data collection period. After that, a practice session was carried out to help participants familiarize with the processes. Altogether, five rounds of small-group student talk were administered as intervention sessions and were recorded by the first researcher with the help of the course instructor (see Table 1). The writing task for the practice session was chosen from the course textbook. The other five writing tasks for the intervention sessions were selected at random from the TEM-4 test battery. Each writing task was structured with an added section of the three requirements mentioned above.

**TABLE 1:** Procedures of the study.

Week	Treatment class	Comparison class	Writing topic
1	Pretest (40 min)	Pretest (40 min)	Whether human brains will get lazy with intelligent machines to do the thinking (2017 TEM-4)
2	Practice session: Small-group student talk for planning (20 min) + individual writing (40 min)	Individual planning (20 min) + individual writing (40 min)	a) Whether college students should be allowed to take cell phones into classrooms (from the course textbook)
3, 5, 7, 9, 11	Intervention sessions: Small-group student talk for planning (20 min) + individual writing (40 min) Posttest	Individual planning (20 min) + individual writing (40 min)	b) Whether college students should hire helpers to clean their dormitories (2010 TEM-4)
			c) Whether private car owners should be taxed for environmental pollution (2011 TEM-4)
			d) Whether English major students should study Mathematics (2014 TEM-4)
			e) Whether tourism will bring harm to the environment (2009 TEM-4)
			Whether it is wise to make friends online (2007 TEM-4)
12		Posttest	Whether human brains will get lazy with intelligent machines to do the thinking (2017 TEM-4)
16	Delayed posttest	Delayed posttest	Whether human brains will get lazy with intelligent machines to do the thinking (2017 TEM-4)

During each round of intervention sessions, students in the treatment class first talked for 20 min about the structured writing task handout and then separated to write a drafted text individually for 40 min. However, students in the comparison class first conducted individual planning for 20 min following the same handout and then proceeded to their individual writing of the task for 40 min. No external sources or help were allowed in either class during each round of intervention. A posttest was given after the fifth round of intervention sessions and a delayed posttest was conducted four weeks after the posttest.

### Data Analysis

In sum, 144 (24 × 2 × 3) drafted texts written by students in both classes were collected from the pretest, posttest and delayed posttest in order to determine what effects small-group student talk had on the quality of argumentation in Chinese tertiary EFL students’ individual argumentative writing.

All the draft texts were rated by two Chinese tertiary EFL instructors with Ph. D. degrees in second language acquisition/applied linguistics, who had no direct involvement in this study. A blind scoring was administered so that both raters had no idea which class texts they were scoring, nor did they know if they were scoring a pre-, post-, or a delayed post-test text. To examine rating consistency and reliability, about 33% of the total texts (48/144) was randomly chosen and scored by the raters. The final score of each written text was the aggregated average value of the scores given by the two raters. Independent rating of the texts resulted in satisfactory reliability with the intraclass correlation being 0.953 for the holistic scores, which could be considered acceptable because it was larger than 0.70 (Multon, 2010). As for the analytic scores in terms of the six elements, the interrater reliability for each element was also adequate (claims, *r* = 0.817; data, *r* = 0.876; counterargument claims, *r* = 0.964; counterargument data, *r* = 0.985; rebuttal claims, *r* = 0.804; rebuttal data, *r* = 0.975).

Statistical analyses using SPSS 26.0 were carried out to address the two research questions. The Shapiro–Wilk tests of normality were run before the analysis to check normality, missing values, and outliers. Results of the Shapiro–Wilk tests revealed that all the data of the current study was normally distributed since the *z*-scores of skewness and kurtosis did not exceed 1.96 (Field, 2009). After that, independent-samples *t*-tests were administered to explore the between-subject differences and see whether there existed any effects of small-group student talk on the quality of argument in Chinese tertiary EFL students’ individual argumentative writing between the treatment class and the comparison class. In the following, one-way repeated measures ANOVAs were carried out to further examine within-subjects differences in each class. Finally, paired samples *t*-tests with a Bonferroni correction which was used to avoid Type I errors would be run if significant changes were perceived from the one-way repeated measures ANOVAs to investigate whether the treatment class significantly showed a larger effect size than the comparison class. During such comparisons, the effect sizes were interpretated using the Cohen’s (1992) criteria which deem that *d* values of 0.20, 0.50, and 0.80 and partial η*2* values of 0.01, 0.06, and 0.14 are respectively considered as small, medium, and large effect sizes.

## Results

### Effects on Overall Quality of Argument

The following Table 2 demonstrates the descriptive data for the subscores of the quality of each argumentative element together with the overall scores of the quality of these elements in Chinese tertiary EFL students’ individual argumentative writing between the treatment class and the comparison class across the three tests. To make sure the baseline conditions of the two classes at the outset of the study, independent samples *t*-tests were applied.

**TABLE 2:** Descriptive statistics for holistic and analytic scores of the quality of argument across tests.

Measures	Class	Pretest		Posttest		Delayed posttest	
				
		Mean	*SD*	Mean	*SD*	Mean	*SD*
Overall	CC	61.25	5.33	63.54	3.70	62.71	3.67
	TC	61.17	4.33	68.00	3.85	66.88	3.93
Claim	CC	4.29	0.690	4.38	0.647	4.29	0.690
	TC	4.08	0.776	4.25	0.676	4.33	0.702
Data	CC	22.04	2.27	22.42	2.02	22.17	1.93
	TC	22.38	2.18	22.67	2.32	22.46	2.21
Counterargument claim	CC	7.33	1.55	8.33	1.05	7.79	1.50
	TC	7.42	1.44	8.96	0.91	8.71	1.33
Counterargument data	CC	16.63	2.45	16.88	2.03	17.08	2.08
	TC	16.79	2.60	18.96	3.03	18.63	2.80
Rebuttal claim	CC	5.88	2.58	6.04	1.49	5.92	2.39
	TC	5.29	1.00	6.88	1.19	5.67	1.34
Rebuttal data	CC	5.08	3.11	5.54	2.21	5.42	1.77
	TC	5.21	2.69	6.21	1.53	6.04	1.37

*CC, comparison class; TC, treatment class; SD, standard deviation.*

The between-subjects results (see Table 3) suggested that students’ performance in holistic and analytic scores of the quality of argument were similar at the time of the pretest (overall, *p* = 0.953; claim, *p* = 0.331; data, *p* = 0.607; counterargument claim, *p* = 0.848; counterargument data, *p* = 0.820; rebuttal claim, *p* = 0.306; rebuttal data, *p* = 0.882). However, significant differences with large effect sizes were found between the treatment class and the comparison class concerning the overall quality of argument in the immediate posttest (*t* = –4.096, *p* < 0.001, *d* = –1.18) and the delayed posttest (*t* = –3.800, *p* < 0.001, *d* = –1.10). Such results indicated that small-group student talk used as collaborative prewriting discussions enabled students in the treatment class to gain higher scores of the quality of argument compared with those in the comparison class.

**TABLE 3:** Between-subject comparisons of holistic and analytic scores on quality of argument across tests.

Measures	Pretest		Posttest		Delayed posttest	
			
	*t*	*p*	*t*	*p*	*t*	*p*
Overall	0.059	0.953	–4.096	0.000**	–3.800	0.000**
Claim	0.983	0.331	0.655	0.516	–0.207	0.837
Data	–0.518	0.607	–0.399	0.692	–0.488	0.628
Counterargument claim	–0.193	0.848	–2.206	0.032*	–2.234	0.030*
Counterargument data	–0.229	0.820	–2.800	0.007*	–2.167	0.035*
Rebuttal claim	1.034	0.306	–2.142	0.038*	–1.339	0.187
Rebuttal data	–0.149	0.882	–1.216	0.230	–1.371	0.177

*p < 0.05; **p < 0.001.

The application of one-way repeated measures ANOVAs showed that the scores of the overall quality of argumentation changed differently over time in both the treatment class [*F*(2,46) = 71.147, *p* < 0.001, $\eta_{\text{p}}^{2}$ = 0.756] and the comparison class [*F*(2,46) = 5.972, *p* = 0.005, $\eta_{\text{p}}^{2}$ = 0.206]. Paired samples *t*-tests and Bonferroni correction (*p* = 0.017) were further employed to better examine the within-subjects differences in each class. Discernible improvement with large effect sizes in the treatment class was observed across the tests (pretest vs. posttest, *p* < 0.001, *d* = –2.23; pretest vs. delayed posttest, *p* < 0.001, *d* = –1.71) and the effect was retained in the delayed posttest (post vs. delayed posttest, *p* = 0.043).

In contrast, statistically significant differences in the comparison class only appeared from the pretest to the immediate posttest (*p* = 0.001, *d* = –0.76). Such differences did not manifest neither from the immediate posttest to the delayed posttest (*p* = 0.148) nor from the pretest to the delayed posttest (*p* = 0.086). The results of these comparisons suggested that small-group student talk was significantly effective with respect to the overall quality of argument in Chinese tertiary EFL students’ individual argumentative writing.

### Effects on Claim

No statistically significant differences concerning the quality of claim were found between the treatment class and the comparison class, including the pretest (*t* = 0.983, *p* = 0.331), the immediate posttest (*t* = 0.655, *p* = 0.516), and the delayed posttest (*t* = –0.207, *p* = 0.837) (see Table 3). The running of one-way repeated measures ANOVAs revealed that students in each class made no significant improvement across time [*F*(2,46) = 1.856, *p* = 0.168, $\eta_{\text{p}}^{2}$ = 0.075], and [*F*(2,46) = 0.069, *p* = 0.934, $\eta_{\text{p}}^{2}$ = 0.003 respectively]. Such results indicated that small-group student talk had no significant effects on the quality of claim in students’ individual writing.

### Effects on Data

Between-subjects comparisons suggested that students in the treatment class achieved a similar performance to those in the comparison class across the three tests (*t* = –0.518, *p* = 0.607; *t* = 0.655, *p* = 0.516; *t* = –0.207, *p* = 0.837 respectively) (see Table 3). The results of one-way repeated measures ANOVAs indicated that the quality of data did not vary significantly across the tests, neither in the treatment class [*F*(2,46) = 1.417, *p* = 0.253, $\eta_{\text{p}}^{2}$ = 0.058], nor in the comparison class [*F*(2,46) = 1.274, *p* = 0.289, $\eta_{\text{p}}^{2}$ = 0.053]. In other words, students in both classes did not perform significantly differently over time concerning the quality of data.

### Effects on Counterargument Claim

Concerning the quality of counterargument claim, students in both classes performed similarly in the pretest (*t* = –0.193, *p* = 0.848). However, the results of between-subjects comparisons demonstrated that students in the treatment class achieved better performance than those in the comparison class in the posttest immediately after the treatment (*t* = –2.206, *p* = 0.032, *d* = –0.64) and in the delayed posttest 4 weeks after the treatment (*t* = –2.234, *p* = 0.030, *d* = –0.64) (see Table 3).

A further analysis using one-way repeated measures ANOVAs showed that the scores of the quality of counterargument claim changed significantly over time in both the treatment class [*F*(2,46) = 21.109, *p* < 0.001, $\eta_{\text{p}}^{2}$ = 0.479] and the comparison class [*F*(2,46) = 5.536, *p* = 0.007, $\eta_{\text{p}}^{2}$ = 0.194]. Within-subjects analysis using the paired samples *t*-tests with Bonferroni correction (*p* = 0.017) indicated a significant effect with a larger size from the pretest to the immediate posttest in the treatment class (*p* < 0.001, *d* = –1.40) compared with that in the comparison class (*p* < 0.001, *d* = –0.85). Such a large size effect was maintained in the delayed posttest in the treatment class (*p* < 0.001, *d* = –0.90). However, no significant improvement manifested in the comparison class, neither from the immediate posttest to the delayed posttest (*p* = 0.073), nor from the pretest to the delayed posttest (*p* = 0.217). Such results suggested that small-group student talk promoted students in the treatment class to produce better quality of counterargument claim in their individual writing across the tests.

### Effects on Counterargument Data

Table 3 demonstrates that no significant differences were found between the treatment class and the comparison class in the quality of counterargument data in the pretest (*t* = –0.229, *p* = 0.820). In contrast, students in the treatment class achieved a significantly better performance than those in the comparison class in the immediate posttest (*t* = –2.800, *p* = 0.007, *d* = –0.81) and the delayed posttest (*t* = –2.167, *p* = 0.035, *d* = –0.63).

To analyze whether the quality of counterargument data changed significantly within each class in the immediate posttest and the delayed posttest, one-way repeated measures ANOVAs were used. Results showed that significant changes across tests were observed in the treatment class [*F*(2,46) = 66.294, *p* = 0.000, $\eta_{\text{p}}^{2}$ = 0.742], while it was not seen in the comparison class [*F*(2,46) = 2.066, *p* = 0.138, $\eta_{\text{p}}^{2}$ = 0.082]. Paired samples *t*-tests with Bonferroni correction (*p* = 0.017) were then applied to further explore the within-subjects differences in the treatment class. The results indicated that small-group student talk enabled students to make progress in the quality of counterargument data with a large effect size from the pretest to the immediate posttest (*p* < 0.001, *d* = –2.07). No significant improvement was found from the immediate posttest to the delayed posttest (*p* = 0.069) and the large size effect of small-group student talk was retained in the delayed posttest (*p* < 0.001, *d* = –1.68). Results of the between-subjects and within-subjects comparisons revealed that small-group student talk was discernibly effective in facilitating Chinese tertiary EFL students to improve the quality of counterargument data in their individual argumentative writing across time.

### Effects on Rebuttal Claim

The treatment and comparison classes achieved similar performance regarding the quality of rebuttal claim in the pretest (*t* = 1.034, *p* = 0.306) and the delayed posttest (*t* = –1.339, *p* = 0.187). However, the treatment class outperformed the comparison class in this measure in the immediate posttest (*t* = –2.142, *p* = 0.038, *d* = –0.62) (see Table 3). The within-subjects analysis using one-way repeated measures ANOVAs showed that the quality of rebuttal claim varied significantly over time in the treatment class [*F*(2,46) = 15.979, *p* < 0.001, $\eta_{\text{p}}^{2}$ = 0.410], but it was not a case in the comparison class [*F*(2,46) = 0.129, *p* = 0.879, $\eta_{\text{p}}^{2}$ = 0.006].

A series of paired samples *t*-tests with Bonferroni correction (*p* = 0.017) were run to further examine the changes across tests in the treatment class. The results indicated that significant differences with a large effect size were discerned in the treatment class from the pretest to the posttest (*p* < 0.001, *d* = –1.12). Such a large size effect of small-group student talk on the quality of rebuttal claim was also seen from the pretest to the delayed posttest (*p* = 0.001, *d* = –0.81). These results suggested that small-group student talk effectively promoted students in the treatment class to produce better quality of rebuttal claim in students’ individual writing and such effects could be sustained in the delayed posttest.

### Effects on Rebuttal Data

There were no statistically significant differences with respect to the quality of rebuttal data between the treatment class and the comparison class across tests (pretest: *t* = –0.149, *p* = 0.882; immediate posttest: *t* = –1.216, *p* = 0.230; delayed posttest: *t* = –1.371, *p* = 0.177 respectively) (see Table 3). The application of one-way repeated measures ANOVAs indicated that students made significant improvement in the treatment class [*F*(2,46) = 3.436, *p* = 0.041, $\eta_{\text{p}}^{2}$ = 0.130], but not in the comparison class [*F*(2,46) = 1.163, *p* = 0.322, $\eta_{\text{p}}^{2}$ = 0.048]. However, a further within-subjects analysis of such effects in the treatment class using the paired samples *t*-tests with Bonferroni correction (*p* = 0.017) revealed no significant changes across the three tests over time (pretest vs. posttest, *p* = 0.032; posttest vs. delayed posttest, *p* = 0.583; pretest vs. delayed posttest, *p* = 0.089 respectively). In this sense, students in the treatment class did not achieve significantly better performance than those in the comparison class across the three tests. These results suggested that small-group student talk had no significant effects on the quality of rebuttal data in Chinese tertiary EFL students’ individual argumentative writing.

## Discussion

In answer to the first research question, which inquired into whether small-group student talk helps enhance the quality of argument in Chinese tertiary EFL students’ argumentative writing, the overall quality of argument in students’ argumentative writing during the pre-, post-, and delayed post-tests was assessed following a holistic scoring rubric developed and validated by Stapleton and Wu (2015). This rubric drew upon Nussbaum et al. (2005); Nussbaum and Schraw (2007), and Qin and Karabacak (2010), which included six elements of argument (claim, data, counterargument claim, counterargument data, rebuttal claim, rebuttal data). The significant distinctions of the holistic scores in both the immediate posttest and the delayed posttest showed that small-group student talk enabled students in the treatment class to gain higher scores of the overall quality of argument compared with those in the comparison class. In other words, small-group student talk did exert positive effects on facilitating the quality of argument through which Chinese tertiary EFL students could improve their argumentative writing performance and enhance their critical thinking skills. Such a finding lends support to previous studies (Nussbaum et al., 2005; Shin, 2008; Pu, 2010; Neumann and McDonough, 2015; McDonough et al., 2019; Li et al., 2020; Li and Zhang, 2021a) in that talking prior to writing has impact on students’ written texts and quality of argument in students’ writing could be effectively facilitated using talking as a scaffolding tool, because talk created opportunities for students to scaffold within each other’s ZPDs (Neumann and McDonough, 2015; McDonough et al., 2019; Li et al., 2020), co-construct their knowledge and experience (Shin, 2008; Pu, 2010; Li and Zhang, 2021b), and draw from ideas and practices they learn with their peers (Nussbaum et al., 2005; Olsen and VanDerHeide, 2020).

Nevertheless, such results have challenged Shi’s (1998) study which claimed that prewriting small-group discussions had no immediate influence on the writing scores and no noticeable effects of pair-group (both teacher-led and peer-led) talk on students’ individual writing were perceived. The possible reason might be the different writing rubrics the two studies adopted. The current study mainly focused on the measurement of quality of argument (i.e., presence of the Toulmin-based argument elements and quality of reasoning), while Shi’s (1998) study conducted a more comprehensive measurement of organization, linguistic, communicative, and argumentative aspects. Using different writing rubrics might potentially lead to inconsistent results when judging the quality of argumentative writing (Plakans and Gebril, 2017). The influence of such a factor is also found in the discordant results between this study and McDonough and De Vleeschauwer’s (2019). Besides, this study has also reported conflicting findings with Mirazi and Mahmoudi’s (2016) which confirmed that individual planning outperformed pair-group collaborative planning in terms of Iranian students’ overall writing ability measured by content, organization, vocabulary use, language use, and mechanics (Jacobs et al., 1981). In addition to the factor of different writing rubrics, various writing tasks and group sizes might be other factors that lead to the discordant results. The current study selected writing tasks from the writing sections of the standard Chinese TEM-4 battery which emphasizes students’ critical thinking and argumentative abilities, while Mirazi and Mahmoudi’s (2016) study used the ones from a TOEFL essay preparation book which concentrated more on students’ reading and listening comprehension as well as summarizing and rewriting abilities. Meanwhile, group sizes (six-student small group VS. pair group) also enable a direct influence on the quality of group discussions and thus cause different results (Burgoon et al., 2002).

With respect to the second research question about whether there exists any difference in the effect of planning with small-group student talk and that without it, this study found that no discernible differences were perceived in the analytic scores either between or within the treatment class and the comparison class with respect to the quality of claim, data, and rebuttal data across tests. However, the statistical analyses did reveal immediate and sustained effects of small-group student talk on the quality of counterargument claim, counterargument data, and rebuttal claim. Counterargument claim was the only element in which students in both classes made significant improvement, but the treatment class demonstrated a larger effect size. In other words, small-group student talk significantly facilitates the quality of argument in students’ argumentative writing by promoting the quality of counterargument claim, counterargument data, and rebuttal claim, which were viewed as parts of the second-level key elements of argument (Zhang, 2018; Zhang and Zhang, 2021a), because a critical thinker ought to reflect different stances and weigh the pros and cons of each stance (Qin, 2020). Also, advanced arguments tended to implement counterarguments and rebuttals (Wolfe et al., 2009; Paek and Kang, 2017) and the presence of opposing views and counterarguments is of central importance to argumentative writing (Rusfandi, 2015).

Concerning the finding that no significant effects were found in terms of claim and data, one possible explanation might include that claim (assertion in response to a writing topic) and data (evidence to support claims) are the most fundamental and preferred elements for learners (Qin and Karabacak, 2010; Qin, 2013, 2020; Liu and Stapleton, 2014; Stapleton and Wu, 2015; Abdollahzadeh et al., 2017; Zhang, 2018). Thus, it does not matter whether students receive the intervention sessions or not. Either way, students would follow the most basic and natural way that they were already quite familiar with to present their claims and data in their individual writing. Another probable reason might be related to the moves of small-group student talk which students follow during their collaborative discussions. It is worth mentioning that students’ talking moves mainly consisted of three steps as suggested in the structured writing tasks. They first expressed each other’s viewpoints of the argumentative writing topic along with corresponding supporting reasons. After that, they began to argue with one another to defend and justify their viewpoints. Finally, they negotiated to decide which ideas and evidence to select and organize into their writing plan. It is obvious that their claims and data were mainly presented during the first move, in which students did not challenge or argue with each other but took turns to give their claims and data until everyone finished; while the counterargument and rebuttal claims and data were largely produced during the second move. In this sense, without negotiation and arguing with each other, the first move in which students’ claims and data were generated only plays a similar role as what individual planning does. This could help explain why students in the treatment class did not outperform those in the comparison class in these two measures.

As for the finding that no significant effects were discerned in terms of rebuttal data between the classes and within each class, one possible reason might be that students in both classes were not familiar with the writing topic. The mean score of rebuttal data in each class is lower than 6 (out of 25), which indicated that due to the lack of relevant topical knowledge, students in both classes barely produced any rebuttal data. As “the interaction between one’s prior knowledge and the content of a specific passage” (Alexander et al., 1991, p. 334), topical knowledge affects the writing performance and shapes the texts in impromptu essay writing (He and Shi, 2012; Zhang and Zhang, 2021b). In this sense, L2 writing instructors are suggested to give students sufficient exposure to materials covering different types of topical knowledge so that students will be familiar enough with the writing topics to align counterargument and rebuttal claims with the corresponding supporting evidence and thus be able to make their viewpoints logically acceptable and effectively persuasive. Such a finding discords with previous studies that attributed the insufficient generation of counterarguments and rebuttals to cultural influences (Xu and Cao, 2012; Paek and Kang, 2017; Wei et al., 2020), because statistically significant differences were found concerning the immediate and sustained effects of small-group student talk on the quality of counterargument claim, counterargument data, and rebuttal claim. Such effects indicated that engaging students in meaning-making talk scaffolded by the Toulmin argument structure can effectively facilitate them to produce desired elements (Rusfandi, 2015).

Regarding the findings that small-group student talk had immediate and sustained effects on the quality of counterargument claim, counterargument data, and rebuttal claim, a possible explanation might be that small-group student talk enabled students to produce increasingly argumentative structural elements with more logically relevant and acceptable evidence. As the basis for developing content for writing, talk is generative and supportive for the development as well as the articulation of ideas for writing prior to the act of transforming the ideas into written text (Parr et al., 2009). In the process of such collaborative talk, students generated alternative viewpoints, provided, and evaluated reasons and clarifications, and negotiated to decide which ideas and evidence to select and organize into their writing plan. Such collaboration and negotiation led them to consider opinions in opposition to their own (or others’) arguments with corresponding supporting evidence, for talk can influence the construction of knowledge around texts and topics and helps students in exploring ideas, informing the argumentative writing that students do in classrooms (Brady, 2018). Therefore, it is not surprising to see that most texts produced in the immediate posttest and the delayed posttest by students in the treatment class included more counterargument claim, counterargument data, and rebuttal claims as well as a wider variety of supporting evidence, such as expert opinions, statistics, examples, personal experiences, common sense, and logical analysis. Such two-sided argument texts offer writers and audiences better opportunities to carry out deeper negotiation, since good or effective arguments are typically expressed with multiple sides (Zhang and Zhang, 2021b).

On the contrary, although students in the comparison class were also provided with the same structured writing tasks, they failed to achieve so in their individual planning. Because without being challenged and arguing with others as their peers did in small-group student talk, they lacked opportunities to collaboratively facilitate complex understanding and co-construct knowledge through meaningful negotiations with others (Winzenried et al., 2017). Correspondingly, their texts were mainly composed of claims and data together with one counterargument claim and one or two counterargument data, which failed to present a certain number of adequate counterargument and rebuttal claims and data, even though good arguments involve counterargument and rebuttal claims and data to augment writing quality (Wolfe et al., 2009). Moreover, texts of the comparison class demonstrated illogical reasoning which are inaccurate and/or irrelevant in terms of quality (Rapanta et al., 2013; Zhang, 2018), mainly in the form of two types—personal experiences and logical analysis. However, in terms of persuasive power, anecdotal evidence is viewed as less effective than expert, causal, and statistical evidence (Hoeken and Hustinx, 2003). Thus, writers’ own personal judgments and experiences are not regarded as strong evidence to support a claim (Zhang, 2018). The lack of alternative or conflicting viewpoints together with the insufficiency of logically adequate evidence indicated that these texts mainly presented one-sided argument or my-side bias argument (Felton et al., 2015). Such texts seemed structurally well-designed, but they were significantly low in terms of argument quality, because this type of argument is regarded as the least sophisticated form of an argument (Rusfandi, 2015).

## Conclusion

The current quasi-experimental study investigated whether employing small-group student talk as collaborative discussions for prewriting planning helped facilitate the quality of argument (measured by claim, data, counterargument claim, counterargument data, rebuttal claim, and rebuttal data) in Chinese tertiary ELF students’ individual argumentative writing. Statistical results of the holistic scores in both the immediate posttest and the delayed posttest showed that small-group student talk facilitated students in the treatment class to achieve higher scores of the overall quality of argument compared with those in the comparison class, which indicated that small-group student talk was effective for promoting the quality of argument in Chinese tertiary EFL students’ individual argumentative writing. Immediate and sustained effects were also found on counterargument claim, counterargument data, and rebuttal claim. Counterargument claim was the only element in which students in both classes made significant improvement, but the treatment class demonstrated a larger effect size. However, no evident effects were perceived regarding claim, data, and rebuttal data across the three tests. Such findings imply that small-group student talk enabled students to achieve collaborative planning for their individual writing, effectively facilitate themselves to produce desired Toulmin-like elements (Rusfandi, 2015), and promote the quality of counterargument claim, counterargument data, and rebuttal claim in their argumentative writing.

These findings imply that the employment of small-group student talk in the Chinese tertiary EFL learners’ writing classroom is beneficial for developing students’ quality of argument in their individual argumentative writing. Therefore, L2 writing instructors are encouraged to provide their students with enough opportunities to engage in such a talk during which they are able to develop sufficient Toulmin-like elements and present good quality reasoning. Besides, given that no immediate and sustained effects of small-group student talk was identified on rebuttal data due to students’ unfamiliar with the writing topics, L2 writing instructors ought to exposure their students to writing materials with a wide range of topical knowledge to mitigate its influence on students’ writing performance (He and Shi, 2012; Zhang and Zhang, 2021b) and help students accumulate sufficient supporting evidence and data.

Despite these findings and implications, this study has certain limitations. Firstly, due to a small sampling size of participants (*N* = 48) in this study, such findings might not be ideal for generalization. Further studies in this vein can amplify the size of sampling to magnify the reliability of the results. Secondly, a repeated writing task for all the three tests (pre-, post-, and delayed post-tests) was used, which might lead to memory issues and influence students’ writing performance. Therefore, different writing tasks for the three tests can be tried in future research. Finally, this study mainly dwelled on a quantitative measurement and analysis of the argument elements and quality, which lacks an in-depth evaluation and interpretation of the features of each element and the advancement process of reasoning. Thus, future studies are suggested to combine qualitative and quantitative analyses for a more thorough and comprehensive understanding of the quality of argument from both the aspects of structural elements and quality of reasoning.

## Data Availability Statement

The original contributions presented in this study are included in the article/Supplementary Material, further inquiries can be directed to the corresponding author/s.

## Ethics Statement

The studies involving human participants were reviewed and approved by the University of Auckland Human Ethics Committee. The participants provided their written informed consent to participate in this study. Written informed consent was obtained from the individuals for the publication of any potentially identifiable data included in this article.

## Author Contributions

HL designed the study, collected and analyzed the data, and drafted the initial manuscript. LZ revised, proofread and finalized the draft for submission as the corresponding author. Both authors contributed to the article and approved the submitted version.

## Conflict of Interest

The authors declare that the research was conducted in the absence of any commercial or financial relationships that could be construed as a potential conflict of interest.

## Publisher’s Note

All claims expressed in this article are solely those of the authors and do not necessarily represent those of their affiliated organizations, or those of the publisher, the editors and the reviewers. Any product that may be evaluated in this article, or claim that may be made by its manufacturer, is not guaranteed or endorsed by the publisher.

## Footnotes

1 The textbook is named *Writing Critically III*—*Argumentative Writing*, which has been included in the list of officially approved textbooks for use in China’s National Standard Textbooks for English Major Students in Tertiary Institutions. The theme-based textbook is specifically designed for English-major undergraduates with orientations in English language and literature, translation, and business English.
