# Supplementary material for: Investigating Effects of Small-Group Student Talk on the Quality of Argument in Chinese Tertiary English as a Foreign Language Learners’ Argumentative Writing
Source: Front Psychol. 2022 Jun 14;13:868045. doi: 10.3389/fpsyg.2022.868045 (PMC9240094; doi:10.3389/fpsyg.2022.868045)
Supplement: Supplementary file 1 [file Data_Sheet_1.pdf]

Appendix A

Writing task for the pre-, post-, and delayed post-tests
(2017 TEM-4) Nowadays, our life is getting a lot simpler and more convenient because of various intelligent machines. However, some people think that our brains will get lazy in a world run by intelligent machines. Write a composition of at least 200 words on the following topic: *With intelligent machines to do the thinking, will our brains get lazy*?
Please follow these three steps to plan for your writing:
(1) Stating a viewpoint to show agreement or disagreement with the writing topic;
(2) Producing and evaluating ideas and evidence that support and oppose the stated viewpoint;
(3) Selecting and organizing ideas and evidence into a writing plan.

Writing task for Intervention Session 1:
(2010 TEM-4) It is recently reported in a newspaper that six students who shared a dorm at a local university hired a cleaner to do laundry and cleaning once a week. And each of them paid her 60 yuan a month. This has led to a heated debate as to whether college students should hire cleaners. Write a composition of at least 200 words on the following topic: *Should college students hire helpers to clean their dormitories?*
Please follow these three steps to plan for your writing:
(1) Stating a viewpoint to show agreement or disagreement with the writing topic;
(2) Producing and evaluating ideas and evidence that support and oppose the stated viewpoint;
(3) Selecting and organizing ideas and evidence into a writing plan.

Writing task for Intervention Session 2:
(2011 TEM-4) With the development of global economy, the number of cars is soaring, which raised pollution problems to our environment. Some governments proposed a "pollution tax" for private car owners in order to protect our environment. Write a composition of at least 200 words on the following topic: *Should private car owners be taxed for environmental pollution?*
Please follow these three steps to plan for your writing:
(1) Stating a viewpoint to show agreement or disagreement with the writing topic;
(2) Producing and evaluating ideas and evidence that support and oppose the stated viewpoint;
(3) Selecting and organizing ideas and evidence into a writing plan.

Writing task for Intervention Session 3:
(2014 TEM-4) An undergraduate of English at a university, in a recent letter to the university's president, complained about the mandatory math classes he had to take. He said that because a language major had little use for math, he would forget all his math lessons soon after taking the required exams. Write a composition of at least 200 words on the following topic: *Should English major students study Mathematics?*
Please follow these three steps to plan for your writing:
(1) Stating a viewpoint to show agreement or disagreement with the writing topic;
(2) Producing and evaluating ideas and evidence that support and oppose the stated viewpoint;
(3) Selecting and organizing ideas and evidence into a writing plan.

Writing task for Intervention Session 4:

(2009 TEM-4) In recent years, tourism has become a booming industry in some areas of China. Many people believe that tourism brings positive effects on economic growth, so we should try our best to promote tourism. However, others complain that tourism has a negative impact on our environment. Write a composition of at least 200 words on the following topic: *Will tourism bring harm to the environment?*

Please follow these three steps to plan for your writing:

(1) Stating a viewpoint to show agreement or disagreement with the writing topic;
(2) Producing and evaluating ideas and evidence that support and oppose the stated viewpoint;
(3) Selecting and organizing ideas and evidence into a writing plan.

Writing task for Intervention Session 5:

(2007 TEM-4) Nowadays, the Internet has become part of people's life, and millions of young people have made friends online. Write a composition of at least 200 words on the following topic: *Is it wise to make friends online?*

Please follow these three steps to plan for your writing:

(1) Stating a viewpoint to show agreement or disagreement with the writing topic;
(2) Producing and evaluating ideas and evidence that support and oppose the stated viewpoint;
(3) Selecting and organizing ideas and evidence into a writing plan.

Appendix B

Analytic Scoring Rubric for Argumentative Writing (Paul Stapleton &Yanming (Amy) Wu, 2015)

<table>
<tr><td rowspan="2">1. Claim(s) (5%)</td><td colspan="2">Score: 5</td><td colspan="3">Score: 0</td></tr>
<tr><td colspan="2">States point(s) of view</td><td colspan="3">Does not state point(s) of view</td></tr>
<tr><td rowspan="2">2. Data (25%)</td><td>Score: 25</td><td>Score: 20</td><td>Score: 15</td><td>Score: 10</td><td>Score: 0</td></tr>
<tr><td>a. Provides multiple reasons for the claim(s), and
b. All reasons are sound/acceptable and free of irrelevancies</td><td>a. Provides multiple reasons for the claim(s), and
b. Most reasons are sound/acceptable and free of irrelevancies, but one or two are weak</td><td>a. Provides one to two reasons for the claim(s), and
b. Some reasons are sound/acceptable, but some are weak or irrelevant</td><td>a. Provides only one reason for the claim(s), or
b. The reason provided is weak or irrelevant</td><td>a. No reasons are provided for the claim(s); or
b. None of the reasons are relevant to/support the claim(s)</td></tr>
<tr><td rowspan="2">3. Counterargument Claim(s)/Alternative Point(s) of View (10%)</td><td colspan="2">Score: 10</td><td colspan="3">Score: 0</td></tr>
<tr><td colspan="2">Provides counterargument claim(s)/alternative view(s)</td><td colspan="3">Does not provide counterargument claim(s)/alternative view(s)</td></tr>
<tr><td rowspan="2">4. Counterargument Data/Supporting Reasons for Alternative Point(s) of View (25%)</td><td>Score: 25</td><td>Score: 20</td><td>Score: 15</td><td>Score: 10</td><td>Score: 0</td></tr>
<tr><td>a. Provides multiple reasons for the counterargument claim(s) /alternative view(s), and
b. All counterarguments/reasons for the alternative view(s) are sound/acceptable and free of irrelevancies</td><td>a. Provides multiple reasons for the counterargument claim(s)/alternative view(s), and
b. Most counterarguments/reasons for the alternative view(s) are sound/acceptable and free of irrelevancies, but one or two are weak</td><td>a. Provides one to two reasons for the counterargument claim(s) /alternative view(s), and
b. Some counterarguments/reasons for the alternative view(s) are sound/acceptable, but some are weak or irrelevant</td><td>a. Provides only one reason for the counterargument claim(s)/alternative view(s), or
b. The counterargument/reason for the alternative view is weak or irrelevant</td><td>a. No reasons are provided for the counterargument claim(s)/alternative view(s); or
b. None of the reasons are relevant to/support the counterargument claim(s)/alternative view(s)</td></tr>
<tr><td rowspan="2">5. Rebuttal Claim(s) (10%)</td><td colspan="2">Score: 10</td><td colspan="3">Score: 0</td></tr>
<tr><td colspan="2">Provides rebuttal claim(s)</td><td colspan="3">Does not provide rebuttal claim(s)</td></tr>
<tr><td rowspan="2">6. Rebuttal Data[a] (25%)</td><td>Score: 25</td><td>Score: 20</td><td>Score: 15</td><td>Score: 10</td><td>Score: 0</td></tr>
<tr><td>a. Refutes/points out the weaknesses of all the counterarguments, and
b. All rebuttals are sound/acceptable
c. The reasoning quality of all the rebuttals are stronger than that of the counterarguments</td><td>a. Refutes/points out the weaknesses of all the counterarguments, and
b. Most rebuttals are sound/acceptable, but one or two are weak
c. The reasoning quality of most rebuttals are stronger than that of the counterarguments, while one or two are equal to that of the counterarguments</td><td>a. Refutes/points out the weaknesses of all the counterarguments, and
b. Some rebuttals are sound/acceptable, but some are weak
c. The reasoning quality of some rebuttals are stronger than that of the counterarguments, while some are weaker than that of the counterarguments</td><td>a. Refutes/points out the weaknesses of some counterarguments, or
b. Few of the rebuttals are sound/acceptable; most of them are weak, or
c. The reasoning quality of most rebuttals are weaker than that of the counterarguments</td><td>a. No rebuttals are provided; or
b. None of the rebuttals can refute the counterarguments</td></tr>
</table>

Note. [a] An implicit requirement of rebuttal data is subsumed under the requirements of row 4 “Counterargument Data”, that is, each piece of rebuttal data should be aligned with each piece of counterargument data in terms of both quantity and logic.
